# Rice quality prediction and assessment of pesticide residue changes during storage based on Quatformer

**DOI:** 10.1038/s41598-024-59816-8

**Published:** 2024-04-21

**Authors:** Tongqiang Jiang, Furong Deng, Wei Dong, Qingchuan Zhang, Peng Liu

**Affiliations:** 1 National Engineering Research Center for Agri-Product Quality Traceability, Beijing Technology and Business University, Beijing, 100048 China; 2 China Food Flavor and Nutrition Health Innovation Center, Beijing Technology and Business University, Beijing, 100048 China; 3 Department of Agricultural Food Standardization Institute, China National Institute of Standardization, Beijing, 100191 China

**Keywords:** Computer science, Sustainability, Nutrition

## Abstract

Rice serves as a fundamental food staple for humans. Its production process, however, unavoidably exposes it to pesticides which may detrimentally impact its quality due to residues. Therefore, it is extremely necessary to monitor pesticide residues on rice during storage. In this research, the Quatformer model, which considers the effects of temperature and humidity on pesticide residues in rice grains, was utilized to forecast the amount of pesticide residues in rice grains during the storage process, and the predicted results were combined with actual observations to form a quality assessment index. By applying the K-Means algorithm, the quality of rice grains was graded and assessed. The findings indicated that the model had high prediction accuracy, and the MAE, MSE, MAPE, RMSE and SMAPE indexes were calculated to be 0.0112, 0.0814, 0.1057, 0.1055 and 0.0204, respectively. These findings provide valuable technical and theoretical support for planning storage conditions, enhancing pesticide residue decomposition, and monitoring rice quality during storage.

## Introduction

China is one of the largest growers and consumers of rice in the world. Rice is the most consumed food crop in China and occupies a very significant location in the dietary structure of the Chinese population. To ensure high rice yields and enhance quality, farmers usually rely on pesticides to control pests and diseases to protect the crop from damage and ensure food safety. However, if pesticides are used inappropriately or irrationally, residues are retained in rice and these pesticide residues can enter the human body^1^. When we consume rice with high levels of pesticide residues, it may negatively affect our health. Several studies have found an association between pesticide residues and several diseases^2^. Among the pesticide residues applied to rice, imidacloprid is one of the most common. Due to its widespread use over a long period of time, it poses a serious threat to human health, and is particularly toxic to adult females^3^. Carbaryl is a carbamate insecticide with a wide range of agricultural uses. Carbaryl residues, on the other hand, pose a significant environmental risk to humans, as well as causing damage to the body itself. Carbaryl is toxic to humans in many ways, damaging DNA and triggering chromosomal aberrations, leading to reproductive abnormalities in human sperm^4^. The main hazard of fenitrothion is the induction of lipid peroxidation and oxidative damage to polyunsaturated fatty acids, proteins and nucleic acids, which affects the function of various organs in animals and humans^5^. Chlorpyrifos-methyl is one of the most widely used organophosphate insecticides in the world and is moderately toxic, inhibits the enzyme acetylcholinesterase^6^ and may also affect cardiac development in early aquatic vertebrates^7^. Deltamethrin is a synthetic pyrethroid insecticide. It is mainly used in grain storage^8^. Deltamethrin triggers fat accumulation^9^, which leads to obesity, as well as toxicity in humans with symptoms such as nausea, vomiting, headache and dizziness^10^. Therefore, in order to reduce pesticide residues in rice during storage and to ensure food safety and quality of rice, we should comprehensively consider suitable storage conditions. During the storage process, pesticide residues in rice will gradually volatilize and degrade. Research has demonstrated that temperature has an impact on the volatility and rate of breakdown of pesticides, while relative humidity affects the hydrolysis reaction of pesticides^11^. However, long-term storage affects the quality of rice, and suitable temperature and humidity conditions may also promote the growth of microorganisms and biotoxins. Therefore, the selection of appropriate storage conditions and techniques is very important to improve the food safety and quality of rice. In the past few years, rice quality has garnered growing attention, with frequent emergence of new concepts such as rice quality and stability^12^. People gradually realize that it is not enough to pursue the growth of rice production, but it is equally important to ensure the safety and quality of rice. As a result, the current research on rice storage primarily emphasizes the technical factors related to temperature and humidity control, pest management, and maintaining rice quality. The field of agriculture has long recognized the significance of rice storage technology, making it an essential area of research. By controlling the temperature and humidity of stored rice, rice losses and quality changes can be reduced.

Kuyu et al.^13^ assessed the effectiveness of various hermetic storage methods and conventional jute and polypropylene bags in preserving rice quality during a 7-month storage period. The results revealed that metal silos and Perdue Improved Crop Storage (PICS) bags were the most efficient in reducing rice nutrient losses. Luo et al.^14^ conducted a study utilizing data from 1202 farming households across 23 provinces in China. The research employed the propensity score matching (PSM) methodology to analyze the influence of advanced storage facilities on household food security. The findings of the study demonstrated that the implementation of modern storage facilities had a profound impact on household food security. These facilities effectively reduced storage losses, extended the shelf life of rice, and minimized the need for pesticides during storage. Pérez et al.^15^ examined the impact of temperature and humidity on pesticide dissipation in soil. They conducted incubation experiments by treating the soil with insecticides and maintaining container temperatures at 278 K, 288 K, and 298 K, while varying humidity levels at 0%, 20%, 40%, 60%, and 100%. The results revealed that the most favorable conditions for the degradation of chlorpyrifos and diazinon in the soil were humidity levels ranging from 100 to 80% of field conditions, coupled with temperatures below 283 K. Interestingly, higher humidity levels were found to accelerate the degradation process of the pesticides. A study by Liu et al.^16^ investigated the impact of seed storage duration and storage temperature on quality parameters of wheat and wheat flour. The study revealed that storing wheat seeds at low temperatures expedited the aggregation of wheat seed proteins and reduced the time required to reach a steady state. Zhao et al.^17^ explored the consequences of temperature on the storage characteristics among various strains of rice and showed that the deterioration of rice quality could be accelerated under high temperature storage conditions. The study by Zhai et al.^18^ used a low-dose electron beam irradiation treatment and stored high-moisture rice for up to 180 days. The findings of the research demonstrated that electron beam irradiation can be a novel, ecologically sustainable, and risk-free approach to improve rice storage. Moreover, Ding et al.^11^ examined the impact of temperature and relative humidity on the degradation patterns of 5 pesticides in wheat and flour. Their results showed that high temperature and high relative humidity accelerated the degradation process of the five pesticide residues. Furthermore, they observed that the degradation profiles and half-lives of these pesticide residues exhibited variations to varying extents in response to different temperature and relative humidity conditions.

In summary, it is important to study the fluctuations in pesticide residue levels in rice during storage to assess the quality of rice. In this research, the impact of temperature and humidity on pesticide residues in rice were combined with Quatformer neural network modeling to predict pesticide residues during storage. Simultaneously, the pesticide residues underwent clustering and analysis using the K-means algorithm to categorise the rice and evaluate its quality. This study provides an important reference for storage personnel to set the temperature and humidity conditions reasonably. By rationally controlling the storage environment, the level of pesticide residues in rice can be reduced and the quality of rice can be improved during storage. At the same time, this also provides important support for the sustainable development of the rice sector.

## Materials and methods

### Data sources

In this research, the effects of temperature and humidity on five pesticide residues over time during rice storage were investigated. The five pesticides were imidacloprid, carbaryl, fenitrothion, chlorpyrifos-methyl and deltamethrin. The experimental setup was as follows: different temperature levels, 25 °C, 30 °C, 35 °C and 40 °C, were set under the condition of maintaining 65% relative humidity to observe the degradation changes of these five pesticides in rice over time. In addition, the study examined the degradation of pesticides in rice over time while varying relative humidity levels at 65%, 70%, 75%, and 80%, while keeping the ambient temperature constant at 25 °C.

For the detection of pesticide residues in rice, we first placed an equal number of rice samples (1 kg) in the artificial climate chamber set up as described above for simulated storage for a period of 30 days, with daily sampling and testing. In order to determine the levels of pesticide residues, the rice samples first needed to be pre-treated, and we chose acetonitrile (chromatographic purity, Merck, Darmstadt, Germany) as the extraction solvent, and used QuEChERS (Quick, Easy, Cheap, Effective, Rugged, Safe) kits (Part No. 5982-5755CH, Agilent, Santa Clara, CA, USA) for rapid extraction and clean-up. Subsequently, reversed-phase high performance liquid chromatography (RP-HPLC) (Waters semi-preparative liquid chromatograph, Waters, Milford, MA, USA) was used for the determination of pesticide residues in rice, and the liquid chromatographic conditions were set as mobile phase acetonitrile:water = 90:10, column temperature 30 °C, flow rate 1.0 ml/min, and 290 nm and 240 nm. 1200 rice samples were finally collected.

The rice grains used in this study were supplied by Jinjian Rice Co. The study complied with plant sampling guidelines. The authors confirm that the plant material was collected in accordance with relevant institutional, national, and international guidelines and regulations.

### A model for assessing the quality of rice based on Quatformer prediction

#### Predictive modeling of pesticide residues in rice based on Quatformer

**Figure 1:**
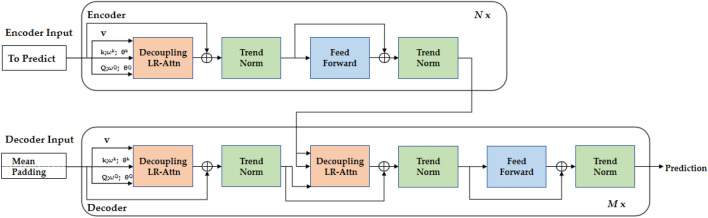
A Quatformer-based modeling framework for pesticide residue prediction in rice grains.

In this paper, we consider the effects of temperature and humidity on pesticide residues in rice by constructing a Quatformer-based prediction model for pesticide residues in rice. We utilized time-series assessment indicators of historical data and environmental factors as model inputs, and correlated these factors with pesticide degradation rates through the Quatformer model. After model training and optimization, we were able to predict the amount of pesticide residue at the next moment under specific environmental factors. Figure 1 depicts the overall structure of the model, where LR-Attn denotes learning-to-rotate self-attention.

The Quatformer^19^ model is a long time series prediction model based on an improved Transformer^20^. The model consists of three main components, which are: 1. quaternion-based Learned Rotational Attention (LRA). LRA incorporates learnable period and phase information to characterize intricate periodic patterns. 2. Trend Normalization. Taking into account the slowly changing characteristics of the sequence representation in the hidden layer of the model, it is normalized to enhance the stability and prediction precision of the model. 3. Global Memory Decoupling LRA. This part achieves linear complexity and ensures the accuracy of the prediction.

The encoder consists of two components, the decoupled learning rotational self-attention and the position feedforward network. The role of decoupled learning rotational self-attention is to capture the complex cycle time patterns of the time series. After these two sub-layers, trend normalization is performed on the results, incorporating gradually changing characteristics into the trend component of the data to provide a more accurate depiction of the sequence in the hidden layer. Unlike the encoder, the Quatformer model inserts a third component into the decoder, namely learning rotational cross-attention, which operates on top of the encoder output.

In the quaternion-based LRA module, the Quatformer model employs a rotated softmax-kernel to fuse the period and phase information of the sequence. The advantage of this approach is that the dot product attention mechanism can be directly applied. By incorporating the LRA module, the model becomes more adept at capturing intricate periodic patterns and extracting relevant information from the time series data. The formulation of the LRA module is shown below.

1 $${\text{SM}}^{{{\textrm{rot}}}} \left( {\varphi \left( {{\text{x}},{\text{m}}} \right),\psi \left( {{\text{y}},{\text{n}}} \right)} \right) = \exp \left( {{\text{Re}} \left[ {\varphi \left( {{\text{x}},{\text{m}}} \right)^{{\textrm{H}}} \psi \left( {{\text{y}},{\text{n}}} \right)} \right]} \right)$$

2 $$\upvarphi \left( {{\text{x}},{\text{m}}} \right) = {\tilde{\text{x}}}{\text{e}}^{{{\textrm{i}}2\uppi \upomega {\text{m}}}} ,\uppsi \left( {{\text{y}},{\text{n}}} \right) = {\tilde{\text{y}}}{\text{e}}^{{{\textrm{j}}2\uppi \upomega {\text{n}}}}$$

where ${\tilde{\text{x}}},{\tilde{\text{y}}}$ is the quaternion form of ${\text{x,y}}$, ${\upomega }$ is the sampling frequency of the rice pesticide residue data, and ${\text{e}}^{{{\text{i}}2\uppi \upomega {\text{m}}}} ,{\text{e}}^{{{\text{j}}2\uppi \upomega {\text{n}}}}$ denote the unit quaternion rotated at the frequency. In addition, ${\upvarphi }$ and ${\uppsi }$ are two different rotation functions in different planes.

LRA relies on ${\text{S}}{\text{M}}^{\text{rot}}$ as a kernel function to learn frequency and phase information in a data-driven manner via a convolutional neural network. In addition, to mitigate model overfitting, the Quatformer model also regularizes the latent frequency and phase. The equation defining the regularization term for a single-layer LRA is shown below (superscripts Q and K are omitted for brevity), where $\omega_{p}^{\left( n \right)} ,\theta_{p}^{\left( n \right)}$ are the n-th terms of $\omega_{p} ,\theta_{p}^{{}}$, respectively. Moreover, $L_{\omega }$ is the l_2_-norm of the difference of $\omega$ and $L_{\theta }$ is the l_1_-norm of $\theta$.

3 $${\text{L}}_{{\upomega }} { = }\frac{{1}}{{{\text{P}}\left( {{\text{N}} - {1}} \right)}}\mathop \sum \limits_{{\text{p = 1}}}^{{\text{P}}} \mathop \sum \limits_{{\text{n = 0}}}^{{\text{N - 2}}} \left( {{\upomega }_{{\text{p}}}^{{\text{(n + 1)}}} - {\upomega }_{{\text{p}}}^{{\text{(n)}}} } \right)^{{2}}$$

4 $$L_{\theta } = \frac{1}{PN}\mathop \sum \limits_{p = 1}^{P} \mathop \sum \limits_{n = 0}^{N - 1} \left| {\theta_{p}^{\left( n \right)} } \right|$$

With the above formula, the ultimate goal of the Quatformer model is to minimize the prediction loss as follows, where $L_{pred}$ denotes predicted loss and $\lambda_{{1}} ,\lambda_{2} > 0$ are hyperparameters.

5 $${\text{L}} = {\text{L}}_{{{\text{pred}}}} + \uplambda _{1} {\text{L}}_{\upomega } + \uplambda _{2} {\text{L}}_{\uptheta }$$

Unlike the layer normalization used by other models, the Quatformer model employs trend normalization. This normalization method learns and represents time series information in the data by imposing a slowly changing feature on the trend component to better normalize the sequence representation in the hidden layer.

In addition, the Quatformer model disentangles attention from global memory, by which global temporal patterns that recur throughout the time series are discovered. The implementation of this step is shown below. Where, M is a sequence of length c used to encode the global temporal pattern of the entire mini-batch, and $\chi$ is a query sequence of length N, and y is a key-value series of length M.

6 $${\text{H}} = {\text{LR-Attn}} \left( {\upchi, \text{M}^{\prime}} \right) \in {\text{R}}^{{{\text{N}} \times {\text{d}}}}$$

7 $$\text{M}^{\prime} = {\text{LR-Attn}} \left( {{\text{M}},{\text{y}}} \right) \in {\text{R}}^{{{\text{c}} \times {\text{d}}}}$$

#### A model utilizing K-means for evaluating rice quality

Leveraging the previous data, we applied the K-Means algorithm to assess the quality of stored rice. To improve the accuracy and effectiveness of assessing rice quality, we have introduced the pesticide residue evaluation index M for rice. The index combines the current pesticide residue values of rice with the predicted residue values, taking into account the impact of potential future pesticide residues on the current rice quality. The formula for the evaluation index M is given in Eq. (8).

8 $${\text{M}} = \left[ {{\text{d}}_{{{\text{k}},{\text{i}}}} ,{\text{d}}_{{{\text{k}},{\text{j}}}} } \right]$$

where ${\text{d}}_{{\text{k,i}}}$ is the current kth pesticide residue of rice, ${\text{d}}_{{{\text{k}},{\text{j}}}}$ is the predicted kth pesticide residue of rice.

In this research, the K-Means algorithm was employed to categorize the risk of rice pesticide residue data M and establish a quality classification framework. The K-Means algorithm is a fundamental and widely utilized clustering technique. The underlying principle is that, with a given value of K and K initial cluster centroids, each data point is assigned to the cluster represented by the nearest centroid of the cluster class. When all the points have been assigned, the centroid of each cluster is then recalculated based on all the points within that cluster, i.e., the average of the sum of the coordinate values of each point is calculated. Next, a loop is executed where points are reassigned and cluster centroids are updated repeatedly until either the centroids remain unchanged or a predetermined number of iterations is reached. Specifically, the steps of the K-Means clustering algorithm are shown below:

1. Initialize the K cluster class centroids.
2. Assign each data point to the cluster that is represented by the centroid of the cluster class that is closest to the data point.
3. Recalculate the location of the centroid for each cluster class.
4. Continue executing steps 2 and 3 until either the positions of the cluster center points remain unchanged or the specified number of iterations is reached.

Through the K-Means clustering algorithm, we are able to classify the rice pesticide residue data into different cluster classes, and then construct the quality grade space of rice in order to realize the assessment of the quality of pesticide residue in rice. Fahlevi et al.^21^ classified 30 rice data brands by K-Means algorithm. The rice brands were finally classified into three categories: very good quality, good quality and poor quality, which reduced the problem of determining the quality of rice. Therefore, in this paper, we will sequentially classify different clusters into different quality classes according to the level of pesticide residue levels in them. Higher quality grade means lower pesticide residue level.

More precisely, the K-Means clustering algorithm has the capability to assign the data points to distinct cluster classes, taking into account their resemblances. For rice pesticide residue data, we can cluster different samples according to their pesticide residue levels and aggregate rice samples with similar residue levels into the same cluster class. By constructing the quality class space of rice, we can associate different clusters with the corresponding residue levels to evaluate the quality of paddy in terms of pesticide residue. The pesticide residue status of rice samples of different quality classes can be derived by counting and analyzing each cluster class.

### Response surface analysis of half-life of pesticide residues in rice grains

In order to further investigate the effects of temperature and relative humidity on the degradation of pesticide residues, a response surface experimental design was carried out based on the results of the one-way test with temperature (A) and relative humidity (B) as the 2 factors and the degradation half-life of the five pesticide residues as the response values, as shown in Table 1.

**Table 1:** Response surface experiment factor level design table.

Level	Temperature (°C)	Relative humidity (%)
− 1	30	70
0	35	75
1	40	80

## Model evaluation metrics

### Methods of statistical testing of pesticide residue data in paddy rice

In order to examine the effects of different storage conditions on pesticide residue levels, the pesticide residue data were examined in this paper using statistical tests. Since the pesticide residue data in this paper showed a monotonically decreasing trend and did not conform to normal distribution, Duncan’s multiple-range test was used in this paper.

Duncan’s multiple-range test is a method used for multiple comparisons after ANOVA, which is mainly used to analyse whether there are significant differences between multiple treatment groups. When using Duncan’s multiple-range test, firstly the means of each group will be calculated and subsequently the difference between the means of each group will be analysed for significance.

### Metrics for evaluating pesticide residue data prediction models for rice

In this paper’s prediction model of rice pesticide residue data, five evaluation metrics were utilized: Mean Squared Error (MSE), Root Mean Squared Error (RMSE), Mean Absolute Error (MAE), Mean Absolute Percentage Error (MAPE), and Symmetric Mean Absolute Percentage Error (SMAPE). These metrics serve as indicators to assess the precision of the prediction model. Their calculation formulas are presented in Eqs. (9–13).

9 $${\text{MSE = }}\frac{{1}}{{\text{n}}}\mathop \sum \limits_{{\text{i = 1}}}^{{\text{n}}} \left( {{\hat{\text{y}}}_{{\text{i}}} - {\text{y}}_{{\text{i}}} } \right)^{{2}}$$

10 $${\text{RMSE = }}\sqrt {\frac{{1}}{{\text{n}}}\mathop \sum \limits_{{\text{i = 1}}}^{{\text{n}}} \left( {{\hat{\text{y}}}_{{\text{i}}} - {\text{y}}_{{\text{i}}} } \right)^{{2}} }$$

11 $${\text{MAE = }}\frac{{1}}{{\text{n}}}\mathop \sum \limits_{{\text{i = 1}}}^{{\text{n}}} \left| {{\hat{\text{y}}}_{{\text{i}}} - {\text{y}}_{{\text{i}}} } \right|$$

12 $${\text{MAPE = }}\frac{{{\text{100\% }}}}{{\text{n}}}\mathop \sum \limits_{{\text{i = 1}}}^{{\text{n}}} \left| {\frac{{{\hat{\text{y}}}_{{\text{i}}} - {\text{y}}_{{\text{i}}} }}{{{\text{y}}_{{\text{i}}} }}} \right|$$

13 $${\text{SMAPE = }}\frac{{{\text{100\% }}}}{{\text{n}}}\mathop \sum \limits_{{\text{i = 1}}}^{{\text{n}}} \frac{{\left| {{\hat{\text{y}}}_{{\text{i}}} {\text{ - y}}_{{\text{i}}} } \right|}}{{\left( {\left| {{\hat{\text{y}}}_{{\text{i}}} } \right|{ + }\left| {{\text{y}}_{{\text{i}}} } \right|} \right){/2}}}$$

In the above formula, n denotes the length of the time series, ${\text{y}}_{\text{i}}$ denotes the actual value, and $\widehat{{\text{y}}_{\text{i}}}$ denotes the predicted value. The formula quantifies the level of disparity between the predicted value and the actual value, with smaller values indicating a better fit of the model. That is, the closer the values of the five indicators MSE, RMSE, MAE, MAPE, and SMAPE are to zero, the smaller the prediction error of the model is.

### Evaluation metrics of rice quality assessment models

In the rice quality assessment model, we chose the silhouette coefficient as an evaluation metric for quality assessment. Silhouette coefficient is a metric used to assess the quality of clusters, which measures the closeness of each sample to the cluster to which it belongs and the separation of the sample from other clusters. To compute the silhouette coefficient, we first calculate the silhouette coefficient for each individual sample. Then, we compute the average of these silhouette coefficients across all samples. The formula for calculation is displayed beneath.

14 $${\text{S}} = \frac{{1}}{{\text{N}}}\sum\limits_{i = 1}^{N} {\frac{b\left( i \right) - a\left( i \right)}{{\max \left\{ {a\left( i \right),b\left( i \right)} \right\}}}}$$

where a(i) denotes the average distance of sample i from other samples in the same cluster and b(i) denotes the average distance of sample i from the nearest other cluster.

By calculating the silhouette coefficient, we can assess the quality of the clustering results. A higher silhouette coefficient indicates that samples within a cluster are closely grouped together and distinct from samples in other clusters. On the other hand, a lower silhouette coefficient indicates a significant overlap between samples and their respective clusters, leading to a less desirable clustering outcome. When the silhouette coefficient is close to 0, it signifies poor clustering results, indicating a high degree of overlap between samples and the clusters they belong to.

## Results and discussion

### Rice quality assessment data set

**Figure 2:**
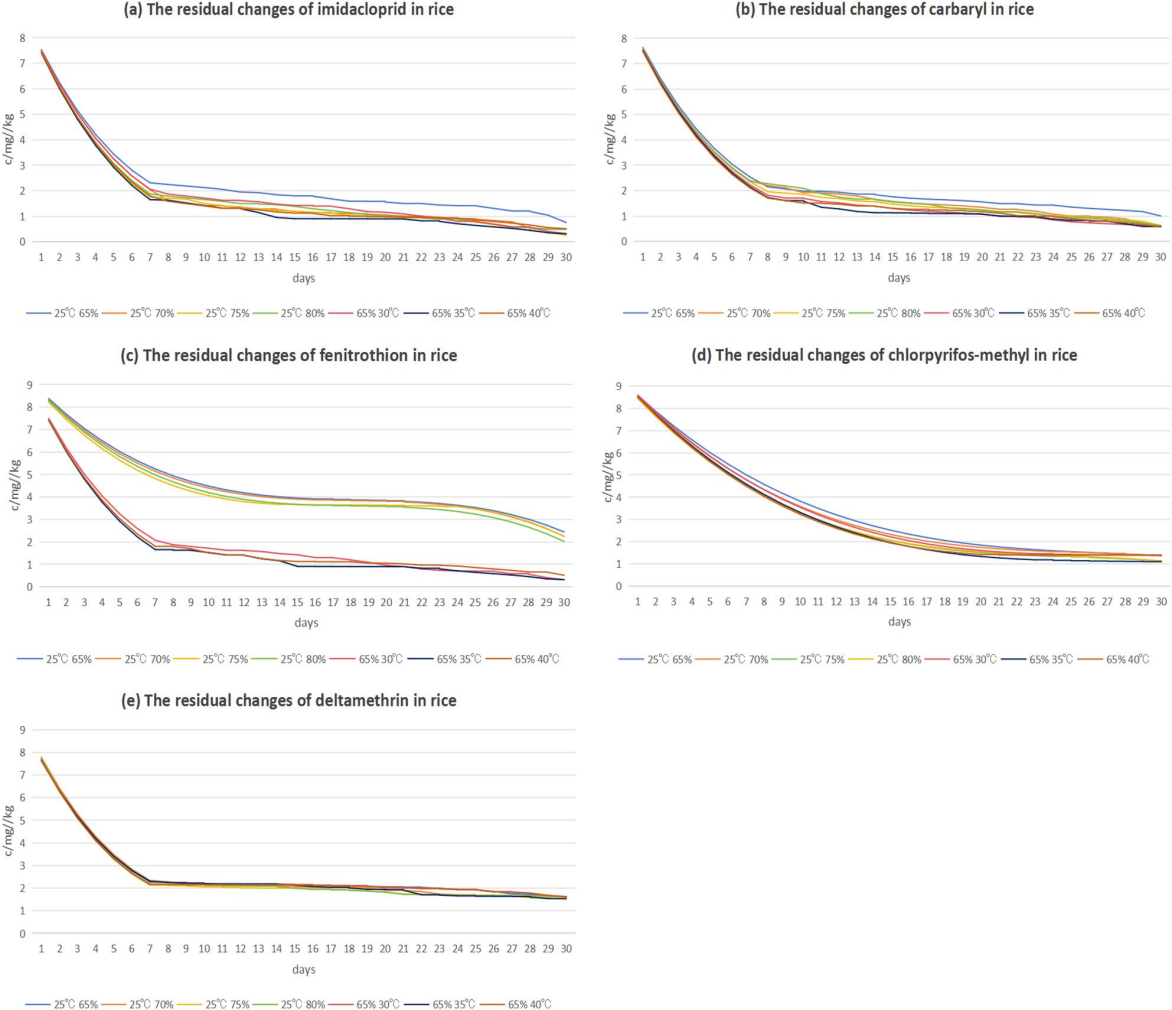
Data set of five pesticide residues in rice.

Based on the characteristics of pesticide residue degradation during grain storage, this study collected residue data of imidacloprid, carbaryl, fenitrothion, chlorpyrifos-methyl, and deltamethrin in rice under different temperature and humidity conditions within 30 days. Figure 2 display the variations in residue levels of these five pesticides in rice during storage at different temperature and humidity conditions.

According to the graph, all pesticide residues in rice grains gradually degrade with the passage of time. Among the five pesticides, the degradation curves of imidacloprid, carbaryl, fenitrothion, and deltamethrin all became flatter after the seventh day, while the degradation curves of chlorpyrifos-methyl became flatter after the fifteenth day. This suggests that chlorpyrifos-methyl cannot be rapidly degraded to lower levels in a short period of time. Besides that, the degradation rate of these five pesticides accelerates as the temperature increases. Nevertheless, when the temperature reaches a specific critical value, the degradation rate reaches the highest point, and then the temperature continues to increase, the degradation rate instead begins to decline. This suggests that as temperature increases, the degradation rate of the five pesticides speeds up. However, when the temperature exceeds a certain point, the degradation rate becomes restricted.

From the trend of the curves presented in the graphs, it can be observed that the shape and slope of the degradation curves of the same pesticide can be affected by temperature and humidity. When the temperature and humidity change, the change of pesticide degradation rate will also cause the shape and slope of the curve to change. An increase in temperature can accelerate the rate of pesticide degradation, so the slope of the curve will be steeper. This means that pesticides are degraded more rapidly at higher temperatures. Conversely, at lower temperatures, the rate of degradation is slower and the slope of the curve will be gentler. In addition, changes in humidity can also have an impact on the degradation process of pesticides. Higher humidity can promote pesticide degradation to proceed, resulting in a greater slope of the curve. This suggests that pesticides are degraded more rapidly in high humidity environments and that the slope of the curve is steeper than in low humidity environments. Therefore, changes in temperature and humidity affect the shape and slope of the degradation curve for the same pesticide. In conclusion, higher temperature and higher humidity accelerate the degradation of pesticide residues and the degradation profile changes with temperature and humidity^11^.

In addition, we used Duncan’s multiple-range test to examine the effect of different storage conditions on pesticide residue levels. Table 2 shows the results using Duncan’s multiple-range test. The number of samples in each group is 30, and groups A to G indicate the environmental conditions in order: 65%, 70%, 75% and 80% humidity at 25 °C and 30 °C, 35 °C and 40 °C at 65% humidity. Different letters in the same row in the table indicate significant differences between treatments (*p* < 0.05).

**Table 2:** Effect of different storage conditions on pesticide residue levels.

Pesticide name	Pesticide residue concentration(mg/kg)						
	Group A	Group B	Group C	Group D	Group E	Group F	Group G
Imidacloprid	2.29 ± 1.56^a^	1.84 ± 1.71^a^	1.74 ± 1.67^a^	1.84 ± 1.66^a^	1.91 ± 1.7^a^	1.65 ± 1.7^a^	1.77 ± 1.67^a^
Carbaryl	2.33 ± 1.6^a^	2.15 ± 1.67^a^	2.07 ± 1.69^a^	2.11 ± 1.69^a^	1.92 ± 1.73^a^	1.86 ± 1.72^a^	1.92 ± 1.68^a^
Fenitrothion	4.46 ± 1.42^a^	4.38 ± 1.42^a^	4.2 ± 1.38^a^	4.18 ± 1.48^a^	1.86 ± 1.72^b^	1.67 ± 1.7^b^	1.81 ± 1.65^b^
Chlorpyrifos-methyl	3.33 ± 2.14^a^	3.2 ± 2.11^a^	2.98 ± 2.14^a^	2.98 ± 2.11^a^	3.15 ± 2.16^a^	2.91 ± 2.22^a^	2.97 ± 2.13^a^
Deltamethrin	2.62 ± 1.45^a^	2.56 ± 1.48^a^	2.45 ± 1.46^a^	2.48 ± 1.47^a^	2.59 ± 1.43^a^	2.53 ± 1.4^a^	2.58 ± 1.4^a^

As can be seen from Table 2, there is a significant difference between groups A, B, C, and D of fenitrothion and groups E, F, and G. This indicates that the degradation of fenitrothion is more susceptible to environmental factors. There was no significant difference between each group for the remaining four pesticides. This may be due to the fact that the chemical structures of these four pesticides are more stable and their degradation is less affected by environmental factors. This indicates that different pesticides have different structures and their sensitivity to environmental factors is also different. Therefore, in order to reduce the residues of these pesticides in rice grains, storage for as long as possible can be considered while maintaining the quality of rice grains.

**Figure 3:**
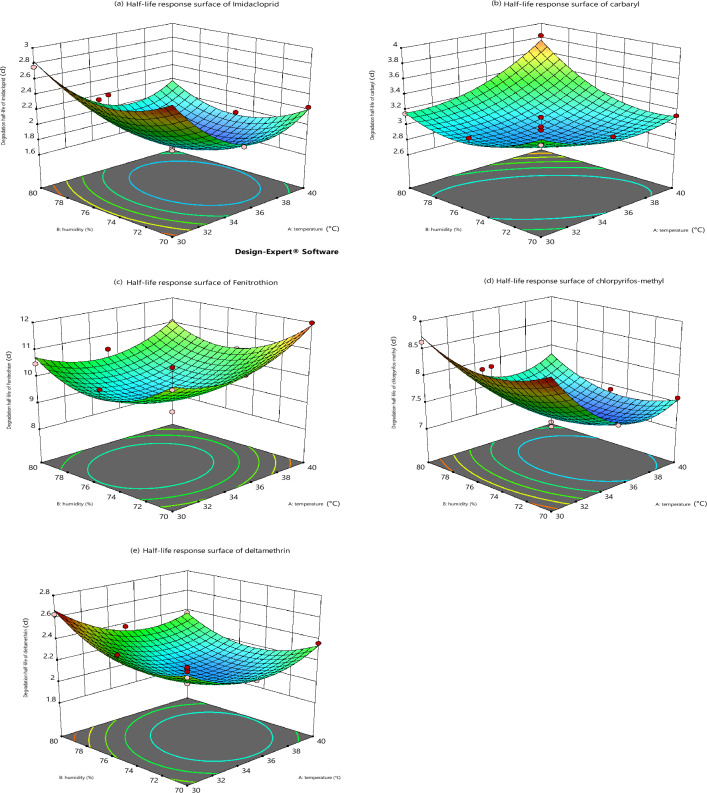
Effect of temperature and relative humidity interaction on degradation of five pesticide residues in rice grains.

Finally, we conducted response surface analyses of the degradation half-lives of five pesticide residues to further investigate the effects of temperature and relative humidity on the degradation of pesticide residues. Based on the previous one-factor experiment, we set three levels of temperature factor as 30℃, 35℃ and 40℃, and three levels of relative humidity as 70%, 75% and 80%, respectively. The half-life response surfaces of the five pesticides are shown in Fig. 3.

As can be seen from Fig. 3, there was an interaction between temperature and relative humidity on the degradation half-life of the five pesticides. With the increase of temperature and relative humidity, the degradation half-life of all five pesticides decreased to different degrees, and when it decreased to a certain value, the degradation half-life of the five pesticides increased instead when the temperature and relative humidity continued to increase. In addition, the slope of the response surface can visualise the sensitivity of the response conditions to the response values. And the steepness of the response surface for the degradation half-life of the five pesticides varied. This suggests that different pesticides have different structures and their sensitivity to temperature and humidity is different^22,23^.

### Predicted results of rice residue during storage

In order to evaluate the performance of our prediction model, we conducted a comparison study with various neural network models, including LSTM, RNN, GRU, and Transformer, using the same dataset of rice pesticide residue quality assessment. Additionally, we assessed their predictive capabilities using five widely adopted model evaluation metrics, namely MAE, MSE, MAPE, RMSE, and SMAPE. Table 3 details the results of these comparisons.

**Table 3:** Comparison of metrics for evaluating models for predicting rice residues during storage.

	MSE	MAE	RMSE	MAPE	SMAPE
LSTM	0.1110	0.2097	0.3331	0.2405	7.1386
RNN	0.0254	0.1421	0.1594	0.1824	6.0516
GRU	0.0216	0.1229	0.1471	0.1654	2.0507
Transformer	0.0201	0.1074	0.1418	0.1514	2.0504
Quatformer	0.0112	0.0814	0.1057	0.1055	0.0204

The accuracy of the Quatformer model can be assessed by its MAE, MSE, MAPE, RMSE and SMAPE values of 0.0112, 0.0814, 0.1057, 0.1055 and 0.0204, respectively. As mentioned earlier, the smaller the value of these evaluation metrics, the smaller the prediction error of the model. Looking further at the results in Table 3, we can see that the difference in the magnitude of the error values between the Transformer model and the GRU model is very small. Furthermore, the error values of both the Transformer and GRU models are considerably lower compared to the error values of the LSTM and RNN models, but the error values are slightly higher relative to the Quatformer prediction model. These results demonstrate that the Quatformer model exhibits better performance in this task. Compared to other models, Quatformer is able to predict and estimate the amount of pesticide residues in rice more accurately. The lower MAE and MSE values indicate that the Quatformer model has a smaller average prediction error, while the lower RMSE values indicate that the model is more robust to prediction errors. In addition, relatively low MAPE and SMAPE values imply that the Quatformer model has a small relative percentage error. These results indicate that the Quatformer model can provide reliable and accurate results for the prediction of pesticide residues in practical applications. Besides, Quatformer has also obtained good prediction results when applied to the prediction of data in meteorology and electricity^19^.

The Quatformer model is able to provide higher prediction accuracy because it employs LRA to better capture complex periodic patterns and related information in the time series, as well as trend normalisation, which is more conducive to dealing with slowly changing features. In addition, the model introduces global memory decoupling LRA to achieve linear complexity, which further ensures the accuracy of prediction. The pesticide residue data used in this paper change slowly for a large part of the time, and the degradation curves of all five pesticides under different environmental conditions are different, so the Quatformer model is used for pesticide residue prediction, which can obtain a higher prediction accuracy.

### Results of rice quality assessment during storage process

**Figure 4:**
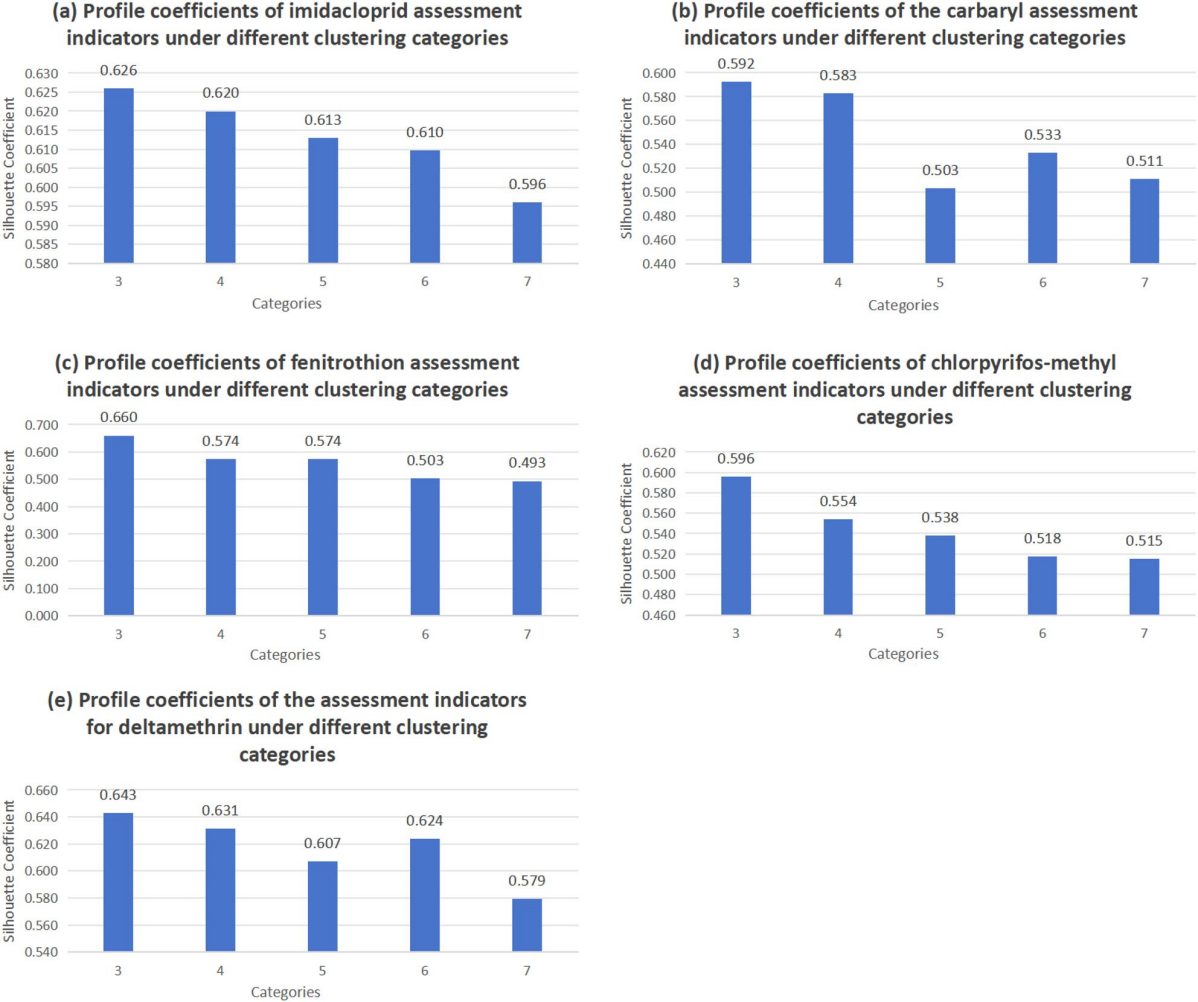
Silhouette coefficients for five pesticide assessment indicators under different clustering categories.

To ascertain the optimal number of clusters, we used the K-Means algorithm for cluster analysis of rice quality in this study. We chose the M-value, an indicator for the evaluation of rice grains under different environmental factors, as a feature for clustering. To evaluate the clustering outcomes, we employed the silhouette coefficient as a metric for assessing the quality. In our experiments, we conducted clustering experiments for five pesticides and set the number of clusters between 3 and 7 according to the actual regulatory needs. By observing the trend of the silhouette coefficient, we were able to determine the optimal number of clusters for clustering. Figure 4 illustrates the silhouette coefficients of the five pesticide assessment indicators under different clustering categories.

By looking at the results in Fig. 4, we can clearly see that the maximum silhouette coefficient is obtained for all five pesticides at a clustering cluster number of 3. This finding suggests that at this particular number of clustering clusters, the rice samples with different qualities are more tightly clustered together in the feature space and the similarity within each cluster is more significant. Based on this result, we can determine the final number of clustering clusters as three in order to categorize rice quality into three classes.

By using the M-value, an indicator for evaluating rice grains under different environmental factors, we can effectively cluster the samples and classify them into high, medium, and low quality levels based on the level of pesticide residues in each category.We derived the concentration intervals of pesticide residues for each grade based on the clustering results of pesticide residues in rice grains, as shown in Table 4. Through Table 4, we can clearly understand the concentration interval of pesticide residues in different quality grades. The lower pesticide residue concentration means the higher quality grade of rice grain.

**Table 4:** Concentration intervals of pesticide residues by grade.

Pesticide name	Pesticide residue concentration(mg/kg)	Quality Level
Imidacloprid	(0, 1.27)	High
	[1.27, 1.64]	Medium
	(1.64, + ∞)	Low
Carbaryl	(0, 1.38)	High
	[1.38, 1.66]	Medium
	(1.66, + ∞)	Low
Fenitrothion	(0, 2.53)	High
	[2.53, 4.08]	Medium
	(4.08, + ∞)	Low
Chlorpyrifos-methyl	(0, 2.17)	High
	[2.17, 3.30]	Medium
	(3.30, + ∞)	Low
Deltamethrin	(0, 1.95)	High
	[1.95, 2.08]	Medium
	(2.08, + ∞)	Low

In the clustering process, we can determine the clustering centers of pesticide residues in rice grains and count the number of samples in each quality level, as shown in Table 5.

**Table 5:** Cluster centers for pesticide residues in rice grains and number of samples for each quality class of rice grains.

Pesticide residue category	di	dj	Sample size	Quality level
Imidacloprid1	0.175094	0.174302	58	High
Imidacloprid2	0.475114	0.377805	44	Medium
Imidacloprid3	0.691503	0.726016	26	Low
Carbaryl1	0.204365	0.229138	58	High
Carbaryl2	0.442228	0.258096	26	Medium
Carbaryl3	0.635743	0.775555	44	Low
Fenitrothion1	0.138968	0.176098	63	High
Fenitrothion2	0.716148	0.454448	51	Medium
Fenitrothion3	0.876827	0.753999	14	Low
Chlorpyrifos-methyl1	0.142882	0.193525	65	High
Chlorpyrifos-methyl2	0.441751	0.391043	41	Medium
Chlorpyrifos-methyl3	0.805059	0.680081	22	Low
Deltamethrin1	0.379217	0.117314	29	High
Deltamethrin2	0.449469	0.529897	35	Medium
Deltamethrin3	0.775338	0.582341	64	Low

Table 5 divides the pesticide residue levels of rice grains into three classes, combining the true pesticide residue values ${\text{d}}_{{{\text{k}},{\text{i}}}}$ and the predicted pesticide residue values ${\text{d}}_{{{\text{k}},{\text{j}}}}$ as the quality assessment indexes of rice grains M. Table 5 presents the clustering centers of pesticide residues, as well as the sample counts for each quality class of rice grains.

By leveraging the data provided in Table 5, we can further analyze the characteristics of pesticide residues in rice grains and their relationship with quality grades. First, by looking at the indicator value of the clustering center, we find that the quality grade of rice grains gradually decreases as the value of this indicator increases. This implies that high clustering center values are associated with higher levels of pesticide residues, which may negatively affect the quality of rice grains.

It is also noted that four pesticides including carbaryl, chlorpyrifos-methyl, imidacloprid, and fenitrothion had more number of samples under high quality grades while only deltamethrin has the most number of samples under low quality grades. This suggests that these four pesticides may be important for the maintenance of rice quality, while deltamethrin may have some problems with quality. Therefore, attention needs to be paid to the spray dosage when using deltamethrin, and biological means of degradation of this pesticide residue can be considered^24^.

Pesticide residues significantly affect the quality of rice grains, so it is crucial to take measures to prevent and control residue problems. Efficient and accurate prediction results using deep learning can assist in the construction of green storage systems during the agricultural production process, thus ensuring rice quality and food security. By predicting the quality of rice and detecting pesticide residues, farmers and processors can take appropriate measures to reduce pesticide use, optimize cultivation and processing, and improve the market competitiveness of rice. Meanwhile, the application of deep learning provides important support and guidance for the rice industry and the quality control of agricultural products^25,26^. By predicting and assessing the quality of rice, agricultural departments can formulate appropriate policies and standards, strengthen supervision and management, and ensure that agricultural products meet quality and safety requirements. The government and related departments can utilize the technical support of deep learning to establish a sound monitoring system and strengthen the supervision of agricultural production, processing and sales, thereby ensuring the quality and safety of agricultural products and promoting sustainable agricultural development.

## Conclusions

Currently, as one of the three major staple foods for human beings, the safety and quality of rice is closely related to human life, and pesticide residues have an important impact on the quality of rice. Pesticide residues in rice are not only hazardous to human health, but may also affect the eating quality of rice and destroy the nutrients in rice. Rice that exceeds the pesticide residue standards will not pass the quality test and thus will not be able to enter the market, which will lead to a decrease in the competitiveness of rice in the market.

Hence, this study primarily emphasized the degradation rate of pesticide residues in rice grains, specifically examining how it is affected by varying storage conditions of temperature and humidity. We focused on five common pesticide residues in rice grains, including imidacloprid, carbaryl, fenitrothion, chlorpyrifos-methyl and deltamethrin. By developing a Quatformer-based model, we were able to forecast the fluctuations in pesticide residue levels during rice grain storage. Meanwhile, with the help of the introduced assessment index M, we combined the pesticide residue values predicted by the model with the actual measured values to assess the quality of rice grains and classify them into different grades. The results showed that changes in temperature and humidity could affect the degradation rate of pesticide residues in rice grains, and both higher temperature and higher humidity could accelerate the degradation rate of pesticide residues in rice grains. By choosing suitable storage environment conditions, the degradation of pesticide residues in paddy can be accelerated, which can help to improve the quality of paddy.

## Data Availability

The data used to support the findings of this study are available from the corresponding author upon request.
